# External Training Load and the Association With Back Pain in Competitive Adolescent Tennis Players: Results From the SMASH Cohort Study

**DOI:** 10.1177/19417381211051636

**Published:** 2021-10-25

**Authors:** Fredrik Johansson, Tim Gabbett, Per Svedmark, Eva Skillgate

**Affiliations:** † Tennis Research and Performance Group, MUSIC, Department of Health Promotion Sciences, Sophiahemmet University, Stockholm, Sweden; ‡ Naprapathögskolan–Scandinavian College of Naprapathic Manual Medicine, Stockholm, Sweden; § Unit of Intervention and Implementation Research for Worker Health, Institute of Environmental Medicine, Karolinska Institutet, Stockholm, Sweden; ‖ Gabbett Performance Solutions, Brisbane, Clayfield, Queensland, Australia; ¶ Centre for Health Research, University of Southern Queensland, Ipswich, Queensland, Australia; # Center for Spine Surgery, Stockholm, Stockholm, Sweden; ** Department of Molecular Medicine and Surgery, Section of Orthopaedics and Sports Medicine, Karolinska Institute at Karolinska University Hospital, Stockholm, Sweden

**Keywords:** workload, acute:chronic workload ratio (ACWR), tennis, adolescent, back pain, injury, cohort study

## Abstract

**Background:**

In young tennis players, high loads on the spine and high training volumes in relation to age are associated with a high lifetime prevalence of back pain. The primary aim of this study was to investigate if accumulated external workload “spikes” in the acute:chronic workload ratio (ACWR) of tennis training, match play, and fitness training, and if high or low workload/age ratio were associated with back pain events in competitive adolescent tennis players. Additional aims were to report the incidence of back pain stratified by sex and level of play and to describe the characteristics of players with back pain.

**Hypothesis:**

Rapid increases in external workload are associated with the incidence of back pain.

**Study Design:**

Cohort study of 198 competitive tennis players, 13 to 19 years, with a weekly follow-up for 52 consecutive weeks.

**Level of Evidence:**

Level 3.

**Methods:**

Accumulated external workload spikes (uncoupled ACWR >1.3), and the workload/age ratio, were time-varying exposures in Cox regression analyses with the outcome back pain (pain intensity ≥2/10 in the lower back and/or in the upper back/neck with a pain-related disability).

**Results:**

For each additional workload spike in tennis training/match play, the hazard rate ratio (HRR) was 1.17 (95% CI, 1.06-1.28) for back pain. The corresponding HRR for fitness training was 1.13 (95% CI, 1.05-1.22). Training workload/age ratio was not related to back pain.

**Conclusion:**

Accumulated external workload spikes of tennis training, match play, and/or fitness training are associated with a higher rate of back pain events in competitive adolescent tennis players.

**Clinical Relevance:**

Back pain is a troublesome clinical problem that may affect the performance of talented young tennis players. Structuring the training schedule to minimize rapid increases (ie, spikes) of training load on a weekly basis may enhance performance and reduce back pain in adolescent tennis players.

Back pain and back injuries are among the most challenging and frequent problems affecting an athlete’s performance.^24^ In a large study of 1114 elite athletes, representing a variety of sports, the lifetime prevalence of back pain was reported to be 88.5%.^10^ In tennis, a repetitive sport with high demands placed on the spine, and high training volumes in relation to age (ie, 17 h/wk at 15 years of age), the lifetime prevalence is as high as 77.5% in young players.^11^ Adolescent athletes may spend more hours per week in sports than years they are old and may therefore be at risk of any injury.^17^ With regard to training load, studies show associations between low back pain and the total amount of training hours performed.^22,25^ Elite junior tennis players are exposed to a combination of high joint loads, frequent repetitions, and physical growth during adolescence, which may contribute to the development of lumbar injuries.^12^ In addition, although asymptomatic at the time of assessment, magnetic resonance imaging investigations of the spine in tennis players have shown a prevalence of radiological abnormalities in the range of 64% to 96% and most frequently in the lumbar region.^1,8,23^ With regard to specific strokes performed, the tennis serve includes lateral flexion of the spine, which transmits load in the lumbar region approximately 8 times greater than those experienced during running.^4^ In addition, since adolescent high-performance tennis players accumulate high training volumes,^10,20^ of asymmetric loading,^28^ it is crucial to monitor the workload of these athletes with respect to the growing spine.^2^

While workload-injury relationships have been extensively studied in many team sports,^3,9,14,19^ few have studied this relationship in tennis.^20,21^ Of the 2 tennis studies performed, the acute:chronic workload ratio (ACWR) was significantly associated with overall injury rate.^20,21^ Although the lumbar region was not specifically studied, it accounted for the second highest proportion of injuries (17.5%) seen in this cohort.^20^ Despite the high incidence of back injuries, there is a paucity of research studies directly evaluating the association between the ACWR and back pain and/or injury across a larger range of sports, including tennis.^27^

Therefore, the primary aim of this study was to investigate if accumulated external workload “spikes” in ACWR of tennis training, match play, and fitness training, and high or low workload/age ratio, were associated with the rate of back pain events in competitive adolescent tennis players. Additional aims were to report the incidence of back pain stratified by sex and level of play and to describe the characteristics of players with back pain.

## Methods

### The SMASH Cohort Study

The longitudinal cohort study SMASH (Shoulder Management and Assessment Serving High Performance) was performed in February 2018 to March 2019 with the main aim of identifying risk factors for shoulder injuries and back pain in competitive adolescent tennis players. A total of 301 players were recruited from the regional and national high-performance program supported by the national tennis association. The players came from all 7 tennis regions across the nation and were 13 to 19 years old. At baseline, a clinical screening was performed and a questionnaire was completed. Informed consent was obtained from the players, and if <15 years of age, players’ legal guardian signed the consent form. Details about back pain and neck pain were measured with a separate questionnaire sent out via an app approximately 1 week after the baseline screening. This distribution was chosen to limit the time required for face-to-face screening. Players were then followed for 52 consecutive weeks with weekly questionnaires sent out each Sunday evening via an app with a reminder 24 hours later if no reply.

### Baseline Measurements

The baseline questionnaire included questions about sex, age, tennis-related factors, history of shoulder problems (Oslo Sports Trauma Research Center Overuse Injury [OSTRC-O]),^6^ athletic identity, general health, sleep, and details about back pain.

The study was performed in accordance with the Declaration of Helsinki, and written consent was received from the regional ethical review board (approval Nos. 2012/1731/2 and 2018/2510).

### Study Population in This Study

**Figure 1.**
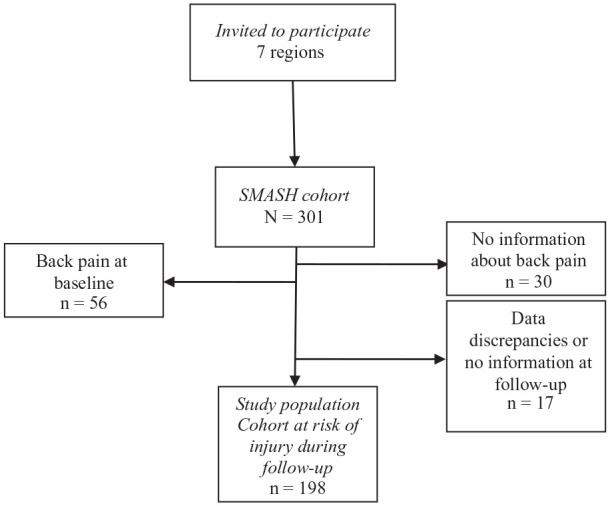
Flowchart describing the inclusion process.

To study a population at risk of developing back pain, only players without back pain (defined as pain in the upper back/neck, the midback and/or the lower back) the preceding 6 months at baseline and those that had answered the follow-up questionnaire were included (n = 198). Figure 1 describes the inclusion process.

### Follow-up Measurements

External workload was measured every week with the following questions: (1) *How many hours and minutes match play have you performed the preceding week?* (2) *How many hours and minutes have you practiced tennis on the tennis court in the preceding week?* and (3) *How many hours and minutes have you had training activities that are not tennis related in the preceding week?* The weekly follow-ups also measured pain intensity in the lower back and/or upper back/neck (11 points Numeric Rating Scale [NRS]), back-/neck pain–related disability (*To what extent has pain in the back/neck affected your athletic performance in the preceding week? [not at all, a little, moderately, to a large extent, could not participate])* complaints/injuries in the shoulder (OSTRC-O),^6^ any acute injury, and number of training days per week. From these weekly follow-ups, information about the time-varying exposures and the time-varying outcome measures was collected.

*Exposure 1: Accumulated external workload spikes*: The weekly uncoupled ACWR using a rolling average were calculated by dividing the sum of training/match hours in the specific week by the mean number of training/match hours in the preceding four weeks. Based on our clinical experience that adolescents do not tolerate large changes in workload, an external workload spike was defined as an ACWR >1.3.
*Exposure 2: Workload/age ratio*^17^: To investigate the association between a high or a low workload/age ratio and the incidence of back pain, a workload/age variable was created with 3 levels: “reference category” ratio = 0.90 to 1.10, “high”—ratio >1.10 (more training/match hours per week than age in years), and “low”—ratio <0.90 (fewer training/match hours per week than age in years). Workload was the mean of the total hours of tennis training/match play and fitness training in the preceding 4 weeks.
*Outcome*: The outcome in the risk analyses was back pain. This was defined as having a pain intensity of 2 or more on the NRS in the lower back and/or in the upper back/neck with a pain-related disability (a little or more).^5,13^ In the risk analyses, only the incidence of a first back pain event was considered. For the estimation of the incidence of back pain over 52 weeks, recurrent events were also considered. A player was classified as having a recurrent event if she/he had at least 1 week without reporting back pain after having been classified as having a back pain event.
*Confounders*: All risk analyses were adjusted for sex, age at baseline, playing level at baseline (national/regional), and number of days with training measured every week in the weekly follow-ups.

The average weekly response rate of the follow-up questionnaires in the full SMASH cohort (N = 301), was 85%, with 51% reporting complete data, 68% answering 90% of the follow-ups, 79% answering 75% of the follow-ups, and 85% answering at least 50% of the follow-ups.

### Statistical Analysis

Last observation carried forward imputation was used to prohibit missing information causing artificial fluctuations in the ratio series. In the risk analyses, the imputed time points were omitted.

The frequency of “external workload spikes” (ACWR >1.3) was calculated separately for tennis training/match, fitness training, and as a combined variable, for the risk analyses, and cumulated over time (from follow-up week 5). These time-varying covariates, and the factor workload/age ratio were used in proportional hazards Cox regression models to calculate the hazard rate ratios (HRRs) with 95% CI. A player was considered to be at risk for a back pain event up until an event occurred, until the player was censored or to the end of follow-up. The proportional hazards assumption was tested using Schoenfeld residuals, and the assumption held for all models but 1.

To address the potential underestimation of the associations between workload spikes and the incidence of back pain using an accumulation of external workload spikes over time, we estimated the association between having had workload spikes or not the week before the week in question (injured/not injured). An analysis adjusted for age, sex, and level of play was performed with generalized estimation equations (GEE) logistic regression with exchangeable covariance structure.

To estimate the association between workload and the incidence of back pain but without using ACWR and spikes, a sensitivity analysis was performed. The regression coefficients from a linear model for the past 4 weeks before the week in question (injury/not) were used to estimate the relationship of the downward/upward slope to the probability of a back pain event on week 5. As it was hypothesized that the relationship of the β-coefficient to injury is not linear, the coefficients were categorized into 5 categories, 1 of which was 0, and the 4 others consisted of the positive and negative coefficients cut into 2 groups from their respective medians. The estimations of the odds of back pain were carried out using the GEE logistic regression with exchangeable covariance structure.

*P* value <0.05 was used to indicate statistical significance. Data management and analyses were done in R (Version 4.0.2; R Core Team, 2020; R Foundation for Statistical Computing) and Stata (Versions 15 and 16; StataCorp 2017 and 2019; StataCorp LLC).

## Results

### Descriptive Analyses

Table 1 describes the baseline characteristics of the full SMASH cohort (N = 301) stratified by back pain at baseline. The mean age was 14.5 years in players free from back pain in the preceding 6 months and 59% of those players were boys. Table 2 describes the back pain characteristics in players that at baseline reported back pain the preceding 6 months (n = 56). The most common pain location in this group was low back pain (80%).

**Table 1.** Baseline characteristics by back pain status at baseline^*a*^

Baseline Characteristics	Back Pain at Baseline (n = 56), Mean (SD)	No Back Pain at Baseline (Risk Cohort) (n = 215), Mean (SD)	*P*	All (n = 271), Mean (SD)
Age, y	14.8 (1.7)	14.5 (2.0)	0.30	14.6 (2.0)
Sex, male, % (n)	52 (29)	59 (127)	0.35	58 (156)
Height, cm	171.7 (11.7)	169.4 (10.7)	0.30	169.9 (10.9)
Mass, kg	61.3 (13.2)	57.8 (12.3)	0.06	58.5 (12.5)
BMI, kg/m^2^	20.5 (2.5)	19.9 (2.5)	0.11	20.0 (2.5)
Passion for sport (AIMS)^*b*^	29.4 (3.1)	29.1 (3.6)	0.57	29.2 (3.5)
Quality of sleep^*c*^	7.8 (1.5)	8.1 (1.7)	0.23	8.0 (1.7)
No. of hours of sleep per night	7.8 (1.3)	8.2 (1.5)	0.07	8.2 (1.5)
General health^*c*^	8.3 (1.9)	8.4 (1.6)	0.69	8.4 (1.7)
No. of matches in year 2017	66.4 (28.7)	65.2 (37.7)	0.82	65.4 (36.0)
Hours per week of tennis training in year 2017	9.8 (3.4)	9.5 (3.8)	0.59	9.5 (3.7)
Hours per week of fitness training in year 2017	4.0 (2.1)	3.7 (2.4)	0.39	3.8 (2.4)
Normal racket tension	23.4 (1.1)	23.4 (1.3)	0.99	23.4 (1.3)
One responsible tennis coach, % (n)	57 (32)	70 (151)	0.07	68 (184)
One responsible fitness coach, % (n)	64 (36)	62 (133)	0.78	63 (171)

AIMS, Athletic Identity Measurement Scale; BMI, body mass index.

a Numbers may differ due to internal missing.

b Sum of total score of 7 items of the AIMS questionnaire (minimum 7, maximum 35) where high scores correspond to a high passion for sport.

c Rated on a numerical rating scale of 1 to 10 where 1 = very bad and 10 = very good.

**Table 2.** Back pain characteristics in players who at baseline reported back pain the preceding 6 months

Back Pain Characteristics (n = 56)	% (n)
Pain locations^*a*^	
Upper back/neck	13 (7)
Midback	25 (14)
Low back	80 (45)
Pain in at least 3 of the preceding 6 months	50 (18)
Pain onset during tennis training or match	61 (34)
Sought care for back problems	68 (38)
Most painful strokes^*a*^	
Serve	63 (35)
Smash	13 (7)
Forehand	27 (15)
Backhand	23 (13)
Volley	0 (0)
Walk over due to back pain^*b*^	21 (12)
Impairment of other activities from back pain	
Sleep	14 (8)
Sit in school	27 (15)
Other physical activities than tennis	34 (19)

a More than 1 answer alternative possible.

b When a player decides not to play a match in a tournament because of back pain.

#### Risk Analysis

An additional external workload spike was associated with an increased back pain incidence in all models (Table 3).

**Table 3.** The associations between workload spikes, and workload/age ratio and the incidence of back pain, presented as hazard rate ratio (HRR) and 95% CI

Training profile	HRR	95% CI
Accumulated workload spikes in tennis training/match play, continuous variable^*a*^	1.17^*b*^	1.06-1.28
Accumulated workload spikes in fitness training, continuous variable^*c*^	1.13^*b*^	1.05-1.22
Accumulated workload spikes in fitness training and/or tennis training/match play, continuous variable^*d*^	1.18^*b*^	1.07-1.30
Workload related to age^*e*,*f*^		
Workload/age ratio 0.9-1.1	1	—
Workload/age ratio <0.9	1.03^*b*^	0.54-1.97
Workload/age ratio >1.1	0.49^*b*^	0.21-1.14

—, no data. ^*a*^χ^2^ test of proportional-hazard assumption: *P* = 0.08.

b Adjusted for age, sex, level of play, and number of days with training/match per week in the preceding 4 weeks.

c χ^2^ test of proportional-hazard assumption: *P* = 0.15.

d χ^2^ test of proportional-hazard assumption: *P* = 0.04.

e The ratio between number of training hours in the preceding 4 weeks and age at baseline.

f χ^2^ test of proportional-hazard assumption: *P* = 0.16.

For each additional workload spike in tennis training/match play, the HRR was 1.17 (95% CI, 1.06-1.28) for back pain. For each additional workload spike in fitness training, the HRR was 1.13 (95% CI, 1.05-1.22) for back pain. Training workload/age ratio was not related to back pain.

As a base for a discussion about the potential limitations of using accumulated spikes in the risk analyses, odds ratio of back pain in players who had workload spikes in comparison with players not having workload spikes was 2.18 (95% CI, 1.20-3.87).

To address the potential limitation of using the ACWR in the risk analyses,^15^ a sensitivity analysis is presented in Table 4. The association between a neutral β-coefficient and a negative and positive β-coefficient, respectively, from the linear model for the past 4 weeks of external workload before the week in question (injury/not) and a back pain event is presented. The odds ratio of back pain in players with a positive slope was 6.76 (95% CI, 2.78-16.46).

**Table 4.** The odds ratio (OR) with 95% CI for back pain, between a neutral β-coefficient and a negative or positive β-coefficient, respectively, and between having had workload spikes or not the week before the week in question (injured/not injured)

	Back Pain	
Workload^*a*^	OR^*b*^	95% CI
Neutral β-coefficient (no slope)	1	—
Negative β-coefficient (slope down)	5.78	2.33-14.33
Slightly negative β-coefficient (slope slightly down)	4.42	1.72-11.36
Slightly positive β-coefficient (slope slightly up)	4.59	1.81-11.66
Positive β-coefficient (slope up)	6.76	2.78-16.46

—, no data. ^*a*^The regression coefficients from a linear model for the past 4 weeks’ external workload (tennis training, match play, and fitness) before the week in question (injury or not) and the estimation of the relationship of the downward/upward slope to the probability of being injured on week 5.

b OR calculated by with generalized estimation equations logistic regression with exchangeable covariance structure adjusted for age, sex, and level of play.

#### Incidence in Tennis Training/Match Play

The incidence of a back pain event per 1000 hours of tennis training/match play and the cumulative number of spikes in the risk cohort (n = 198) for all players and stratified by sex and level of play are presented in Table 5. In total, 85 players (43%) had at least 1 back pain event during the follow-up period in weeks 5 to 52. This corresponded to an incidence of a first back pain event of 0.91 (95% CI, 0.85-0.97) per 1000 hours for all players.

**Table 5.** Incidence of back pain and the cumulative number of spikes for all and stratified by sex and level of play (n = 198).

	All n = 198	National n = 35	Regional n = 163	Boys n = 114	Girls n = 84,
Type of Incidence	n, Incidence (95% CI)	n, Incidence (95% CI)	n, Incidence (95% CI)	n, Incidence (95% CI)	n, Incidence (95% CI)
Incidence of first back pain, per 1000 tennis/match play hours	85, 0.91 (0.85-0.97)	15, 0.69 (0.52-0.92)	70, 0.98 (0.94-1.01)	45, 0.79 (0.69-0.90)	40, 1.10 (0.81-1.50)
Incidence of back pain over 52 weeks, per 1000 tennis/match play hours	197, 2.11 (1.83-2.42)	37, 1.70 (1.23-2.35)	160, 2.23 (1.91-2.60)	104, 1.82 (1.51-2.21)	93, 2.55 (2.08-3.13)
Total number of cumulative spikes in fitness training and/or tennis training/match play during 52 weeks					
0, n (%)	22 (15)	4 (11)	18 (11)	17 (15)	5 (6)
1-10, n (%)	120 (61)	28 (80)	92 (56)	66 (58)	54 (64)
>10, n (%)	56 (28)	3 (9)	53 (33)	31 (27)	25 (30)

Furthermore, in total, 197 back pain events, including recurrent events of back pain, were reported during the follow-up period in weeks 5 to 52. This corresponded to an incidence per 1000 hours of tennis training/match play of a back pain event of 2.11 (95% CI, 1.83-2.42) for all players.

## Discussion

### Main Results

The purpose of this investigation was to examine if external workload spikes were associated with the incidence of back pain in adolescent competitive tennis players. Additionally, the incidence of back pain stratified by sex and level of play and the characteristics of players with back pain was reported. Furthermore, we examined how the volume of hours of sports per week in relation to the athletes’ age was associated with back pain. The main findings of this study indicated that external workload spikes in tennis training, tennis match play, or fitness training are associated with a higher incidence of back pain in competitive adolescent tennis players. Comparisons of our results with others are difficult since training load, sample size, follow-up time, and classification of injury differ.

We did not find any associations between the workload/age ratio and the incidence of back pain, indicating that in terms of injury risk, adaptation of workload to age may not be important in this age span. However, a slight trend toward a decreased risk was seen in the group with a training volume in hours >110% of their age. From a clinical perspective, 1 possible explanation can be that a higher training volume develops the musculoskeletal system, which may protect the players from back pain. On the other hand, previous authors reported higher training volumes in tennis players than the participants’ age (ranging between 113% and 203%) and at the same time, a high lifetime prevalence of either back pain and/or lumbar spine problems.^10,20^ Therefore, coping with high training volumes may be one of the biggest challenges for adolescent players. In summary, this ratio needs further investigation to clarify whether age related to workload is a risk factor for back pain in adolescent tennis players.

### Incidence of Back Pain and Workload Spikes Stratified for Sex and Playing Level

Female tennis players revealed a higher incidence of back pain than boys in our study, even though the boys reported a higher training load. However, in view of the cumulative number of spikes in training load, there were no differences between the sexes. Therefore, the explanation for the difference between sexes may be other factors previously discussed in the literature such as earlier maturation in girls, anatomical characteristics, and/or the impact from the menstrual cycle. It may also be that female players are less robust when it comes to muscular fitness (ie, strength) and/or that the technical skills are not as developed, especially in the high loaded service motion.^18^ In summary, the literature on sex differences in the incidence of back pain in athletes is inconclusive, making specific recommendations difficult.

With regard to playing level, national players reported a lower incidence of back pain, despite their higher training volume per week. These results are in conflict with other studies on back pain in athletes where a positive association between back pain and training volume were found.^10,11^ A possible explanation for the contradictory results is the consistency in training volume from week to week performed by most (80%) of the national players in our study. In addition to training consistency, a cumulative number of spikes (>10 per year) was only seen in 9% of the national players compared with 33% of the regional players. Furthermore, national players have better biomechanical efficiency in the different strokes (ie, serve, forehand, backhand) and therefore spare the lumbar spine from high loads.^18^ From a clinical perspective, it is plausible that building a high chronic external workload in combination with a sound technique may be beneficial for the player in terms of musculoskeletal resilience and adaptation to training.

### Characteristics of Players With Back Pain

The SMASH cohort was divided into players free from back pain (n = 215) and players reporting back problems (n = 56) in the preceeding 6 months (yes/no) (see Table 1). The 21% of players reporting back pain in the preceding 6 months in our SMASH cohort is much lower than previously reported,^10,11^ although because of methodological differences direct comparisons are difficult. There were no differences between the cohort free from back pain and the group with back pain at baseline for age or anthropometry. Furthermore, no difference was seen in the score of passion for sport assessed by the Athletic Identity Measurement Scale or in general health. However, the players with back pain tended to report slightly lower quality and quantity of sleep, compared with players without back pain.

With reference to training volume, including both fitness and tennis, only a minor difference of plus 0.6 hours per week was seen in the back pain group. Strength and conditioning has become increasingly highlighted over the past decade and the role of the fitness coach has become more important. In the present study, no difference were found between groups with or without a responsible fitness coach. However, the back pain group less frequently (57%) had a responsible tennis coach compared with the cohort free from back pain (70%). In the back pain group, 63% reported their back pain to be mostly related to the serve, with the most painful area being the lumbar region, (80%) which is in line with the literature.^7,12^ From a clinical perspective, the importance of having a responsible tennis coach should be highlighted with regard to teaching the players a correct and an efficient serve technique.^7,19^ All differences between players with and without back pain in the cross-sectional comparison (Table 1), might be affected by the fact that the players had experienced back injury and/or pain in the preceeding 6 months.

### Methodological Discussion

A strength of the present study is the longitudinal analyses regarding a first event of back problems of a cohort free from back pain at baseline.^26^ The methods used to measure the outcome entails a low risk of misclassification of the outcome; an NRS for pain intensity and the verbal rating scale to measure pain-related disability are among the most used in research and are suggested to be appropriate for measuring changes in functional status and pain in patients with acute low back pain.^13^ The follow-up rate of the weekly questionnaire was on average 85%, which is relatively high. Furthermore, considering that the study population was the vast majority of the adolescent tennis players at the regional and national level, this study has a low risk of selection bias and good external validity. Another strength is that we considered a number of confounders in the risk analyses. However, when the exposure is time varying such as in the present study, ideally the potential confounders should also be time varying. Only the factor “number of days training per week” was time varying, mainly because of the limited space in the follow-up questionnaires to measure other time varying confounders such as sleep and stress. Therefore, there is a risk of unmeasured and residual confouding.

There are further limitations of this study that warrant discussion. First, a potential underestimation of the associations may arise from the fact that we used an accumulation of external workload spikes over time as a time-varying covariate in the analyses. To address this, we also estimated the association between having had workload spikes or not the week before the week in question (injured/not injured). The odds for a back pain event in players who had workload spikes in comparison with players not having workload spikes were higher even without accumulating the spikes. Second, a potential limitation is that we modeled a ratio (ACWR) in risk analyses.^16^ As a sensitivity analysis, we estimated the relationship of the regression coefficients from a linear model for the past 4 weeks before the week in question (injury or not) to the probability of being injured on week 5, indicating that stable workload during the preceding 4 weeks was protective for injury in week 5 (Table 4). This stability can be a proxy for having a low number of workload spikes, and therefore the results support the results found regarding accumulated workload spikes. Third, an ACWR threshold >1.3 was chosen for the categorization of a so-called workload spike. This threshold was chosen based on our clinical experience that adolescents do not tolerate large changes in workload. However, another cutoff may have yielded a slightly different result. Finally, while the SMASH cohort study was relatively large, the statistical power for some of the analyses was low. Considering these strengths and limitations, we believe our results are valid.

### Clinical Relevance

Back pain is a troublesome clinical problem for young talented tennis players because of its limiting effect on performance. Structuring the training schedule to minimize rapid increases (ie, spikes) of training load on a weekly basis may enhance performance and reduce back pain in adolescent tennis players.

## Conclusion

Accumulated external workload spikes of tennis training, match-play, and/or fitness training are associated with a higher rate of back pain events in competitive adolescent tennis players.
